# Scaled deployment of *Wolbachia* to protect the community from dengue and other *Aedes* transmitted arboviruses

**DOI:** 10.12688/gatesopenres.12844.3

**Published:** 2019-08-13

**Authors:** Scott L. O'Neill, Peter A. Ryan, Andrew P. Turley, Geoff Wilson, Kate Retzki, Inaki Iturbe-Ormaetxe, Yi Dong, Nichola Kenny, Christopher J. Paton, Scott A. Ritchie, Jack Brown-Kenyon, Darren Stanford, Natalie Wittmeier, Nicholas P. Jewell, Stephanie K. Tanamas, Katherine L. Anders, Cameron P. Simmons

**Affiliations:** 1 Institute of Vector-Borne Disease, Monash University, Clayton, VIC, 3800, Australia; 2 College of Public Health, Medical and Veterinary Sciences, James Cook University, Cairns, QLD, 4878, Australia; 3 Division of Epidemiology and Biostatistics, School of Public Health, University of California, Berkeley, USA; 4 Centre for Statistical Methodology, London School of Hygiene and Tropical Medicine, London, UK; 5 Department of Medical Statistics, London School of Hygiene & Tropical Medicine, London, WC1E 7HT, UK

**Keywords:** Dengue, World Mosquito Program, Eliminate Dengue, Aedes aegypti, mosquito release, community engagement

## Abstract

**Background**: A number of new technologies are under development for the control of mosquito transmitted viruses, such as dengue, chikungunya and Zika that all require the release of modified mosquitoes into the environment. None of these technologies has been able to demonstrate evidence that they can be implemented at a scale beyond small pilots. Here we report the first successful citywide scaled deployment of *Wolbachia* in the northern Australian city of Townsville.

**Methods**: The *w*Mel strain of *Wolbachia* was backcrossed into a local *Aedes aegypti* genotype and mass reared mosquitoes were deployed as eggs using mosquito release containers (MRCs). In initial stages these releases were undertaken by program staff but in later stages this was replaced by direct community release including the development of a school program that saw children undertake releases. Mosquito monitoring was undertaken with Biogents Sentinel (BGS) traps and individual mosquitoes were screened for the presence of *Wolbachia* with a Taqman qPCR or LAMP diagnostic assay. Dengue case notifications from Queensland Health Communicable Disease Branch were used to track dengue cases in the city before and after release.

**Results**: *Wolbachia* was successfully established into local *Ae. aegypti* mosquitoes across 66 km ^2^ in four stages over 28 months with full community support.  A feature of the program was the development of a scaled approach to community engagement. *Wolbachia* frequencies have remained stable since deployment and to date no local dengue transmission has been confirmed in any area of Townsville after *Wolbachia* has established, despite local transmission events every year for the prior 13 years and an epidemiological context of increasing imported cases.

**Conclusion**: Deployment of *Wolbachia* into *Ae. aegypti* populations can be readily scaled to areas of ~60km ^2^ quickly and cost effectively and appears in this context to be effective at stopping local dengue transmission

## Introduction

A growing body of evidence shows that the *w*Mel strain of *Wolbachia*, when introduced into *Aedes aegypti*, reduces the mosquito’s ability to transmit key human viruses such as dengue ^1^, Zika ^2, 3^ and chikungunya ^4, 5^, and this reduction is estimated to have the potential to significantly reduce disease transmission in affected communities ^6^. The World Mosquito Program (formerly known as the Eliminate Dengue Program), a not-for-profit consortium, has demonstrated previously that, after small-scale releases, the *w*Mel strain of *Wolbachia* can be established and maintain itself within isolated *Ae. aegypti* populations around the city of Cairns in Australia ^7, 8^. Subsequent pilot releases have also shown that *Wolbachia* can be established in contiguous urban habitats ^9^. In this report, we present the results of the first large-scale deployment of *Wolbachia* across Townsville, a medium-sized city in northern Australia with a population of ∼187,000 residents.

Our goals for this work were to demonstrate that large scale deployment of *Wolbachia* was possible ^10^, that it could be done quickly and efficiently at low cost, and that it was acceptable to communities. In addition, while not designed as a clinical trial, it also provided an opportunity to examine a time series of observational data on dengue transmission, for 13 years before deployment and four consecutive dengue transmission seasons since deployment began.

## Methods

### Community engagement

One of the key objectives of the Townsville project was establishing a community engagement framework that could be suitably scaled for a citywide deployment and could be used cross-culturally for future deployments. Previous deployments in Cairns had relied on obtaining individual consent from community members for the release activities, an approach that was unsuitable for the required scaling. Instead we developed a Public Acceptance Model (PAM) for our engagement that formed the basis for obtaining community support for the research activities. The PAM was based on a set of Public Participation Principles described in Table 1.

**Table 1.** Public participation principles of the World Mosquito community engagement approach.

Principle	Measure of Success
*Respectful* Caring for and heeding the interests and concerns of others	1. Issues raised by people are treated as valid and properly considered
*Inclusive* Making an effort to include everyone within its scope	2. Efforts are made to include all people with a potential interest in the project in project communications 3. People are able to nominate their interest in being included in the project communications
*Transparent* Being clear, open, and not hiding anything	4. Project information relevant to community understanding and interest is readily available and kept up to date
*Responsive* Showing that requests or concerns have been heard and trying hard to accommodate them	5. Commitments made by project personnel are met 6. Public requests for information are responded to promptly 7. Concerns raised are listened to and efforts taken to resolve them
*Honest* Telling the truth, not trying to deceive or allowing untruths to prevail	8. All communications about the project are factual and cover the information of potential interest to people 9. Information is presented in appropriate forms and languages so that all interested people can understand

The PAM consisted of four key components:

1. **Raising awareness** by providing information to residents and key stakeholders about the program. These activities included face to face meetings, media events, stalls at community markets, community presentations utilising existing community networks such as community associations, information kiosks in public spaces, traditional and electronic mail outs of information letters and deployment coverage updates, a public billboard and newspaper advertising, a school outreach program and social media incentive program.
2. **Quantitative surveys** that measured community awareness and acceptance conducted by an external market research company, Compass Research. Each telephone survey was undertaken at roughly six monthly intervals, the first survey being undertaken in March 2014 prior to our community engagement activities starting in the city and each involved 200–600 participants ( Table 2).
3. **An issues management system** that allowed community members to easily contact the program with questions or concerns and have them addressed by program staff typically within 24 hours of receipt. This also allowed residents to opt out of direct participation if they had concerns.
4. **A community reference group** that consisted of respected community members from key stakeholder groups and included representation from Townsville City Council, Queensland Health, the local indigenous community, the Defence Force, local business, community development and environmental groups, the tourism sector and the education sector. The reference group’s primary function was to independently review our activities to ensure that we had carried out our engagement in accordance with our commitments and stated Public Participation Principles ( Table 1). The reference group was tasked to evaluate our activities and make a recommendation to the program management that community engagement had been sufficient for releases of mosquitoes to commence. Before releases began this group met monthly; after releases started they continued to meet every 6–8 weeks. The secondary functions of this group were to test and comment on the suitability of engagement materials and approaches, and to provide the program with feedback on community sentiment towards the program and identify potential issues that might require a proactive response. The reference group was also kept regularly updated on the latest results of the program.

**Table 2.** Results of telephone surveys seeking to understand community awareness and support for the program.

	Jul 2013 (stage 1 area) n=300	Jan 2014 (stage 1 area) n=300	Sept 2014 (stage 1 area) n=600	Dec 2014 (stage 1 area) n=300	Oct 2015 (stages 2, 3, 4 area) n=600
Awareness (unprompted)	17%	29%	49%	51%	62%
Awareness (prompted)	52%	59%	69%	80%	62%
Awareness via media (TV, radio, paper)	N/A	69%	66%	65%	78%
Very Comfortable or Comfortable with the research	91%	85%	89%	95%	92%
Very Comfortable or Comfortable with community mosquito releases	N/A	N/A	N/A	95%	87%

### Rearing

In order to establish the colony for release, wild mosquito eggs were collected from ovitraps set at 49 sites across Townsville and used to produce a wildtype colony. Material from this colony was stored as dried eggs and amplified only as required. Amplification of material from this colony was limited to F3 for use in outcrossing during colony maintenance. For stage 1 of the Townsville releases, eggs were produced from insectaries at Monash University, Melbourne or James Cook University, Cairns and shipped to the Townsville field office. For stages 2–4 all mosquito material was produced at Monash University.

The wildtype colony was backcrossed for three generations to a laboratory line infected with the *w*Mel strain of *Wolbachia* ^11^. This new colony, TSV *w*Mel.f was continuously maintained in order to produce ∼800,000 eggs per week. To maintain the material during mass production, the TSV *w*Mel.f line was divided into two distinct colonies: ‘broodstock’ and ‘release material’. The ‘broodstock’ colony was reared under the more relaxed conditions described in 12 but kept at 26°C. Its purpose was to produce eggs for amplification and production of the ‘release material colony’. In order to prevent inbreeding, 10% wildtype males (from the same wildtype material as was used for backcrossing) were added to each generation of the ‘broodstock’. The purpose of the ‘release material’ colony was to produce eggs for release; it did not provide any material for the next generation in the laboratory. In order to facilitate mass production, the ‘release material’ colony was maintained as described for the broodstock with the following modifications. No wild material was added to the ‘release material’ colony. Once eggs were hatched, first instar larvae were aliquoted into 500 ml plastic cups at a ratio of 150–180 larvae/400 ml of water. The larvae were fed once with half a fish food tablet (Tetramin Tropical Tablet, Tetra Holding (US) Inc., Germany) until pupation. Larval rearing cups were transferred to adult cages for emergence once 60% of larvae had pupated. Cages were stocked at a rate of ∼600 adults per (30 X 30 X 30cm) cage.

For both colonies, females (5–7 days old) were fed with human blood (Monash University Human Ethics approval CF11/0766 – 2011000387). They were provided the bloodmeal by introducing the arm of a volunteer into the selected cage. Females were fed until repletion (usually 10–15 minutes). Females were fed once per week, for one or two weeks depending on requirements. For safety, only one bloodfeeder was used per cage and bloodfeeders who showed any signs of fever or who were taking antibiotics were excluded.

Three 22 cm oviposition strips of red cotton duck cloth were placed in each cage three to five days after bloodfeeding. Oviposition strips were removed from cages four days later, and sandwiched between two double layers of 3mm thick kitchen sponge that had been covered with a single layer of paper towel, covered with a 3mm thick Perspex sheet and placed on a rack. Eggs were allowed to dry this way in an 80%RH controlled-temperature room for up to 24 hours before being placed in humidified containers. The humidity in these containers was maintained at ∼80%RH by providing a saturated KCl solution inside the containers.

After the oviposition strips had been dried, the density of eggs/cm on each strip was estimated to determine the length of egg strip to be cut for subsequent use in Mosquito Release Containers (MRCs). Eggs were then shipped to the Townsville field lab.

Hatch rate was tested for every batch of eggs produced. Matched sets of eggs were taken from a number of strips and photographed to assess desiccation and overall quality of the eggs. One portion of each matched set was shipped to the release site, and one set kept at the rearing facility. Once the eggs reached the release site, both sets of eggs were counted, hatched, and hatch rate determined by counting larvae. Hatch rate of 70% or above was considered acceptable. If hatch rate fell below 70%, the cause of this drop was investigated. In most cases, the cause was determined to be due to fluctuating environmental conditions or to slight changes made to the drying procedure, which was altered slightly throughout releases.

*Wolbachia* infection frequency was also tested each week of production. 80 females and 80 males were screened from each broodstock cohort using diagnostic qPCR as described below. If *Wolbachia* frequency fell below 97% in any broodstock cohort, the eggs from their resultant ‘release material colony’ would not be used for release, however this issue never arose.

The James Cook University rearing strategy differed slightly from the Monash rearing strategy. A single colony of ∼10,000 Townsville *w*Mel-infected *Ae. aegypti* sourced from Monash was created in a semi-field flight cages ^13^ in the Tropical Medicine Mosquito Research Facility located at James Cook University in Cairns. Based upon experience with earlier releases, we assumed that there is a loss of ∼50% of the colony per week. The colony was therefore refreshed with 2500 males and 2500 females each week. We also conducted backcrossing to maintain genetic diversity by adding males (10% of cage male population) sourced from an uninfected wildtype Townsville colony (< F4). To prevent introduction of wild females and potential loss of *Wolbachia* infection into the colony, we only added males. This was achieved by placing suspected male pupae based on size into cups of 10; any cups containing emerged females were discarded.

Females (5–7 days old) were fed with human blood on volunteers (JCU Human Ethics H4907). They were provided the bloodmeal by introducing 5 volunteer blood feeders into the field cage 3–5 times/week who let mosquitoes feed for 10 minutes. For safety bloodfeeders were screened at every feed for possible exposure to dengue infected mosquitoes using a questionnaire to access travel history, and their temperature was taken to detect fever. Any volunteers with fever, a possible exposure to dengue infected mosquitoes or who were taking antibiotics were excluded for a minimum of 2 weeks.

Eggs were harvested from partially flooded 10 L buckets containing 26 × 30 cm strips of red felt cloth placed in the semi-field cage. A perspex template 31cm in length with 12 1-cm holes drilled into it was placed over the cloth to limit oviposition to the exposed 1 cm area of the ovistrip. The ovistrips were collected 3 times/week, embryonated and dried three days later. Once removed from the cages, oviposition strips were placed on moist paper towel in a sealed plastic container, after 3 days the lid of the sealed container was removed and the eggs were allowed to dry this way in an 80%RH controlled temperature room for up to 24 hours before being placed in humidified containers. The humidity in these containers was maintained at ∼80%RH by providing a saturated KCl solution inside the containers. The cloth was then cut into individual eggstrips containing a single egg clump that could be deployed into egg release containers in the field. The number of eggs on each eggstrip was estimated by using reference photographs of eggstrips with known egg numbers as visual guides for fast estimation.

### Mosquito releases

**Figure 1.**
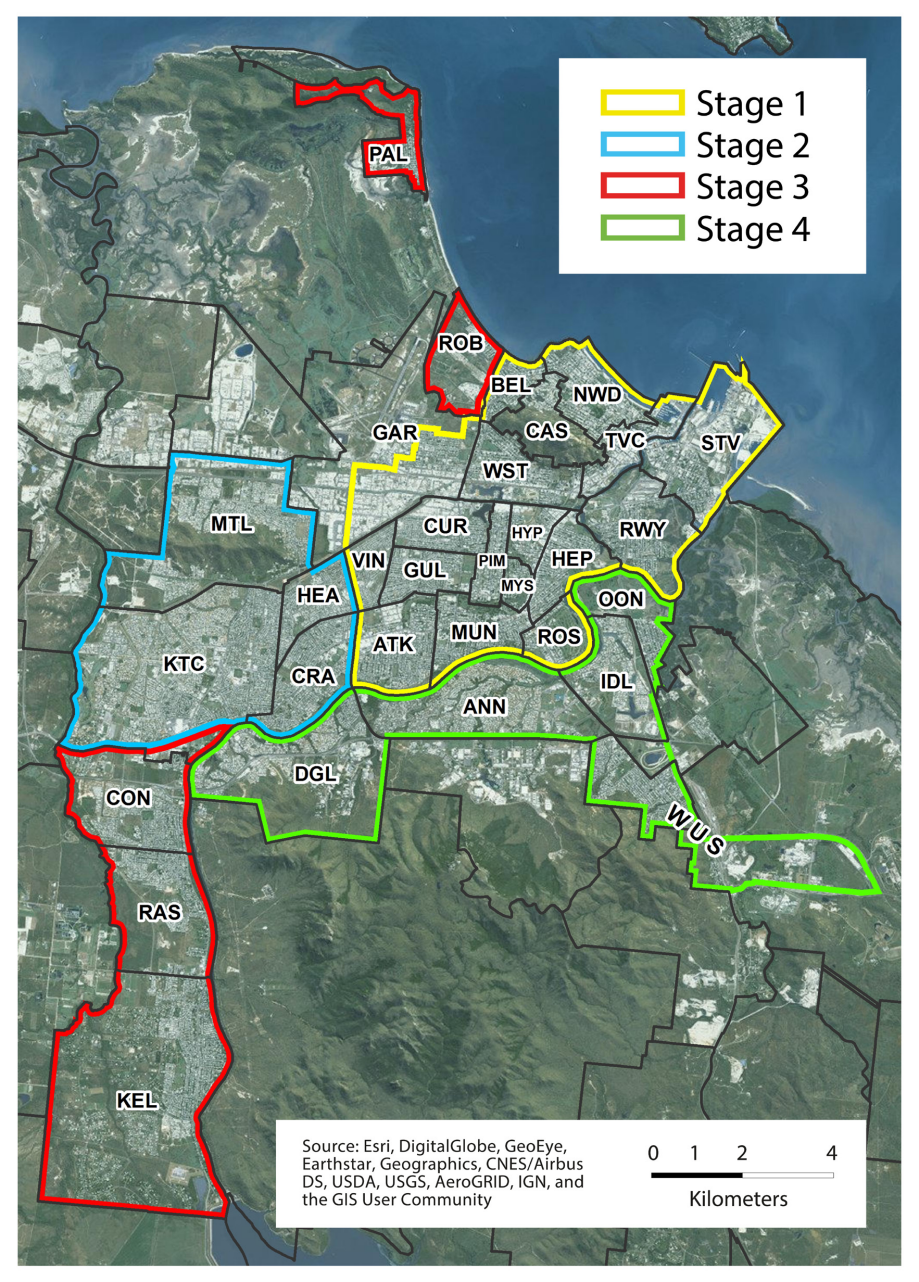
Release site. Map of Townsville city showing the boundaries of the four release stages.

The municipal area of Townsville is ∼190km ^2^. However, within this area there were many areas where releases did not take place due to the lack of suitable *Ae. aegypti* habitat. Releases were restricted to residential and business areas within the city where *Ae. aegypti* breeding was likely to occur. This resulted in the actual area for release being reduced to approximately 66km ^2^ to effectively cover the city. The release program was divided into four stages ( Figure 1).

**Figure 2.**
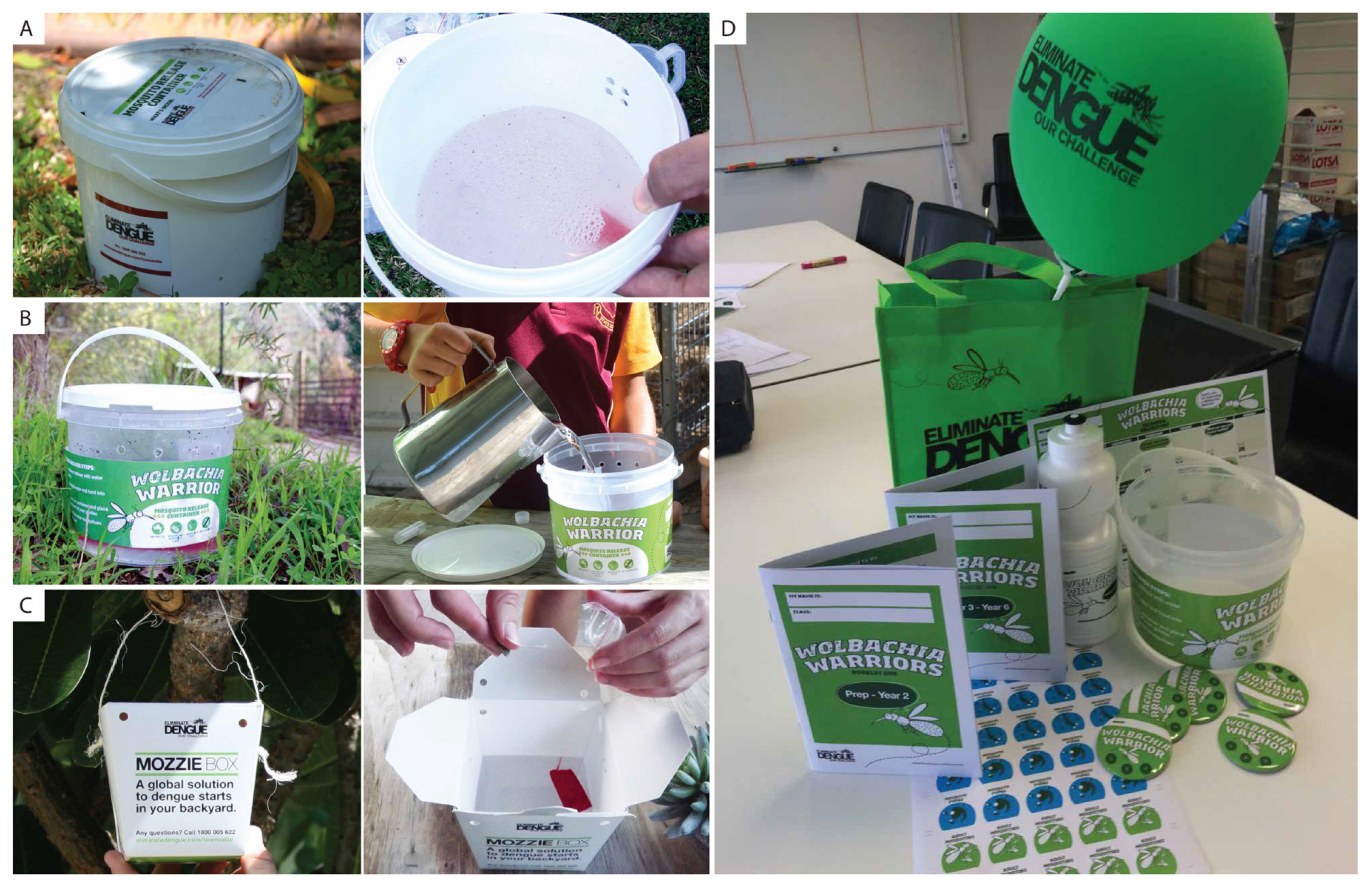
Release containers. Photos illustrating different mosquito release containers used in the deployment. ( **A**) Bucket mosquito release containers (MRCs) used in stage 1 releases ( **B**) Clear bucket MRC used in Wolbachia Warriors school program in stage 1 ( **C**) Mozzie Box MRC that was used in stages 2–4 ( **D**) Material given to school children as part of the Wolbachia Warriors program.

Stage 1 covered a release area of 20km ^2^ and included the suburbs with known highest dengue transmission risk: South Townsville, Railway Estate, North Ward, Townsville City, Belgian Gardens, Castle Hill, West End, Garbutt, Currajong, Vincent, Gulliver, Aitkenvale, Mundingburra, Rosslea, Hyde Park, Pimlico, Mysterton and Hermit Park. In this stage, all releases were undertaken using bucket mosquito release containers (MRCs). These were 2.3L white polypropylene pails with lid (Peopleinplastic, Australia), with top 164mm diameter, base 145mm diameter, and height 147mm. Each bucket had four 6mm holes drilled 20mm apart in a square pattern in the side ( Figure 2A). The inside of each bucket was roughened with sandpaper to allow mosquitoes to rest upon emergence. Into each bucket MRC was placed an egg strip containing approximately 100 viable eggs (estimated from hatch rate QA), 5 (summer) or 6 (winter) wafers of Aqua One vege wafer fish food (Aqua Pacific, UK) and 1L water. More food was provided in winter and the servicing cycle for these buckets was extended from 2 to 3 weeks to allow for longer emergence times.

 Bucket MRCs for stage 1 were placed by program staff in outdoor shaded areas at approximately 20% of all residential properties in a roughly evenly spaced arrangement in each suburb. They were serviced every two weeks by tipping out the water, cleaning the bucket and adding new food, water and eggs. An average of 88 adult mosquitoes were released from each bucket MRC in stage 1. Releases continued in each suburb until the frequency of *Wolbachia* in samples of field-caught mosquitoes from that suburb was above 50% for two consecutive weeks. For stage 1, it required between 7 and 19 weeks of releases for each suburb to reach that target

Stages 2, 3 & 4 covered release areas of 18, 18 and 10 km ^2^ respectively, and included the following suburbs. Stage 2: Cranbrook, Heatley, Kirwan/Thuringowa Central and Mount Louisa; stage 3: Condon, Pallarenda, Rowes Bay, Rasmussen and Kelso; stage 4: Idalia, Oonoonba, Wulguru/Stuart, Annandale and Douglas. Releases for these later stages did not rely on program team members to place all release containers. Instead, they utilised strategies that directly involved the community, such as the use of school students, direct community release, or through collaboration with local businesses. Releases for these stages also used Mozzie Box MRCs ( Figure 2C) which consisted of a 775ml Food Pail (Detpak, Australia) without handle, and with measurements top 104×92mm, base 79×61mm, height 104mm. Four 5mm holes were punched into each MRC – one hole approximately 1cm from the top right and top left corners of each long-side face of the box. Each Mozzie Box MRC received 100 viable eggs (estimated from hatch rate QA), 4 (summer) or 5 (winter) wafers of Aqua One vege waters, and 400ml tap water. Mozzie Box MRCs were not re-used.

 In stages 2–4 the goal was again to place MRCs at 20% of residences in the release area. This was done by using community engagement activities to identify participants who would agree to host an MRC. In areas where there were large spatial gaps in participation, the program team would then supplement coverage by visiting additional houses in these areas and obtaining consent to leave MRCs with residents at these locations. Finally, in the last two suburbs of stage 3 (Kelso & Rasmussen) and across stage 4, releases of adult mosquitoes ^7^ were used to fill in gaps in MRC coverage.

During the 28 months of the release phase (stages 1–4), a total of approximately 4 million mosquitoes were released. Releases were undertaken with regulatory approval from the Australian Pesticides and Veterinary Medicines Authority (APVMA permit numbers PER14797 and PER82947).

### School releases

The Wolbachia Warriors Program was developed both as a tool to engage children and their parents and make them aware of the program, and as an alternative channel to release mosquitoes. Five different primary schools were selected to run the program over the duration of the Townsville project. One school participated in each stage except for stage 2 where two schools participated. In total 943 students aged 6–12 participated in these programs.

School children were provided with a bucket MRC in stage 1 as used in operational releases in stage 1 but made of clear plastic to encourage student observation ( Figure 2B) and Mozzie Box MRCs in stages 2–4 ( Figure 2C), complete with mosquito eggs, food, instructions, a calendar to track progress, a magnifying glass, a badge for participation, and an educational booklet tailored for either lower (grade P-2) or upper primary (grade 3–6) students ( Figure 2D). Each student was expected to undertake three consecutive releases with their MRC over a six-week period.

Materials were distributed at the schools by program communication and engagement staff, who gave presentations encouraging participation prior to each of the three mosquito release cycles. Students were asked to use their calendar to record the progress of the mosquito life cycle in their MRC, and to return it to program staff at the end of the release.

### Direct community release

In these releases, a Mozzie Box MRC was provided directly to residents who set it up and reared the mosquitoes themselves at their place of residence. In stages 2-4, more than 6,000 households directly participated in establishing *Wolbachia* by managing their own release container. Almost half of these participants contacted the program team to receive an MRC, which was subsequently delivered to their house. The remaining participants were recruited through doorknocking, or through other recruitment methods such as community groups. Additional Mozzie Box MRCs were distributed through large local employers including the City Council, Telstra, The Townsville Hospital, James Cook University and Queensland Nickel. More than 200 people participated in these programs.

### Quality assurance procedures

In stage 1, program staff checked 5–10% of all bucket MRCs to determine whether the bucket had failed or not, and if not to count pupal skins to obtain an estimate of adult emergence from which they could estimate release rates. In stage 2 – 4, a random selection of 5–15% of all MRCs were checked to determine if they were set correctly. Larvae, pupae and pupal skins were counted to estimate emergence rates in these stages (accounting for potential delayed development of mosquitoes at time of QA due to community members setting up MRCs later than day of delivery). This approach was supplemented in stage 3 with additional sentinel buckets that were set and checked by staff to determine average emergence rates. These data were then used to adjust numbers of eggs placed in MRCs.

### Monitoring

Up to 172 Biogents Sentinal (BGS) traps were progressively rolled out across stage 1 during releases at a density of approximately 8 BGS traps per km ^2^. For stages 2–4 the BGS trap density was reduced to 4 per km ^2^, resulting in 74 – 115 traps being deployed per stage. Exact trap numbers fluctuated due to operational considerations (i.e. trap location no longer suitable, trap broken or missing, community request for trap to be removed or resident moved etc.).

**Figure 3.**
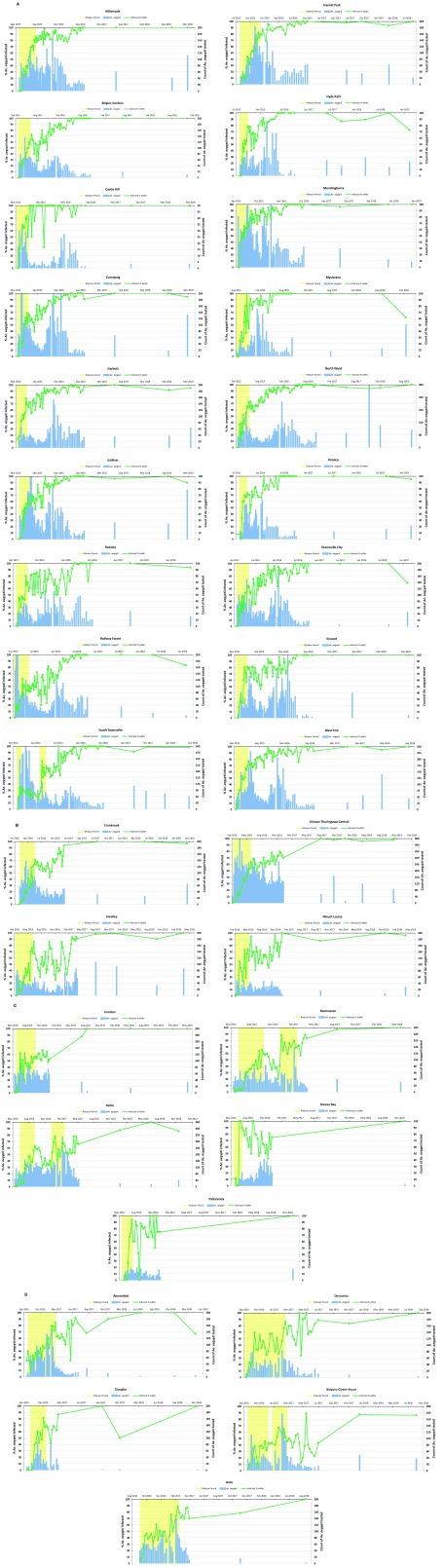
Wolbachia establishment by suburb. For each stage ( **A**– **D**) and suburb *Wolbachia* frequency is plotted against time. Yellow shading indicates periods when releases were undertaken. Bars show the number of mosquitos captured in Biogents Sentinel (BGS) traps and tested for *Wolbachia*.

Samples from BGS traps were collected weekly and returned to the field office for morphological identification. *Ae. aegypti* samples were stored in 70% ethanol and shipped to Monash University for diagnostic determination of *Wolbachia* infection status. After Feb 2016, samples were collected fortnightly instead of weekly as occurred in stage 1 until traps were finally removed from each suburb ( Figure 3). Sites then moved to long-term annual monitoring.

### Diagnostics

Adult *Ae. aegypti* samples collected from BGS traps in the field were screened for *Wolbachia* using Taqman qPCR on a Roche LightCycler 480 using a qualitative assay for presence or absence of *Wolbachia* as previously described ^14^ but with the replacement of the Cy5-BHQ3 fluorophore-quencher pair in the *w*Mel probe with the fluorophore-quencher LC640-IowaBlack (Integrated DNA technologies) to remove some of the Cy5 probe instability observed under varying light and ozone levels ^15^.

From Septemeber 2018 diagnostics was done by LAMP. LAMP primers (Integrated DNA Technologies, Singapore) were designed to detect the *wsp* gene from *w*Mel and *w*MelPop-CLA strains using the software LAMP Designer 1.02 (PREMIER Biosoft International). Individual reactions consisted of 2X WarmStart® Colorimetric LAMP Master Mix (New England BioLabs, Cat# M1800S), primers according to the manufacturer recommendation ( Table 1), and 1 μL of target DNA in a total reaction volume of 17 μL. Reactions for individual samples were performed in 96-well PCR plates (LabAdvantage 96-well PCR plates, full skirt, clear). Plates were incubated in a thermocycler (BioRad C1000) at 65°C for 30 minutes then held at 12°C until scoring. Within one hour of incubation, colour changes of individual samples were recorded. Primers were as follows FIP 5’ TGTATGCGCCTGCATCAGCTTCGGTTCTTATGGTGCTAA, BIP 5’ GCAGAAGCTGGAGTAGCGTTGTGTCATGCCACTTAGATGG, F3 5’ TGATGTAACTCCAGAAGTCA, B3 5’ CTTATTGGACCAACAGGATCG, LpF 5’ AGCCTGTCCGGTTGAATT, LpB 5’ CAGTCTTGTTATCCCAGTGAGT.

### Dengue case notification data

Dengue is a notifiable disease in Australia, which mandates clinicians and laboratories to report confirmed and suspected cases to local health authorities (See Queensland Dengue Management plan). Non-identifiable data was provided by Queensland Health Communicable Diseases Branch for all laboratory-confirmed and clinically diagnosed (probable) dengue cases with illness onset between 1 January 2000 and 31 March 2019, extracted from the Notifiable Conditions System (NOCS) on 3 July 2018. Case notifications within the Townsville local government authority were tabulated by month of illness onset and history of recent overseas travel during the 3 – 12 days prior to illness onset; a variable that is routinely captured in case notifications based on interview by public health teams (see 16 for interview protocol). The suburb of residence of four locally-acquired dengue cases notified in Townsville since *Wolbachia* deployments commenced in October 2014 was determined from situation reports published by the local public health unit.

### Interrupted time series analysis

Negative binomial regression was used to model monthly counts of locally-acquired dengue cases (January 2001 – March 2019) in aggregate *Wolbachia*-treated and untreated areas of Townsville. The regression model was fitted in Stata (SE version 14.2, StataCorp, TX) using generalised estimating equations, with epidemic year (September – August) as a cluster variable to account for temporal autocorrelation in the monthly case counts, adjusting for monthly imported dengue cases (any vs none) and season (wet: June – November vs dry: December – May), with a population size offset. A binary intervention variable was included in the regression model to distinguish the pre- or post-intervention status of each area in any given month, the coefficient of which provided the estimate of intervention effect (incidence rate ratio). Robust standard errors were used.

Populations were derived from mesh blocks (Australian Bureau of Statistics, 2016) aggregated to the boundaries of each operational release area. Aggregate treated and untreated areas (and their resident populations) were dynamic over time, with the treated area in any given month defined as the total area where *Wolbachia* deployments had been completed to date. Cases’ location, for the purpose of classifying *Wolbachia* exposure status in this analysis, was determined using address information from the Townsville Public Health Unit (PHU) operational database. The address classified in the PHU dataset as the probable location of dengue acquisition was used where available (95/468 cases, 20%); if unavailable then the primary residential address was used (248/468, 53%). For 108/468 cases (22%) the address in the operational database was not designated as ‘acquired’ or ‘residential’, and for the remaining 17 cases (6%) no address was available in the PHU database, and the suburb of residence from the NOCS dataset was used to define the case’s location.

### Ethical considerations and consent

Ethics approval for human blood feeding mosquito colonies in Melbourne was issued from Monash University CF11/0766 a 2011000387 (Rearing mosquitoes using blood from human volunteers). All volunteers (no children involved) provided written consent.

In Cairns, Human Ethics approval for bloodfeeding (H6286) was provided by Human Research Ethics Committee, James Cook University. All adult subjects provided informed oral consent (no children were involved). Names of subjects providing oral consent were recorded in writing.

Townsville community mosquito releases were covered under Monash University ethics: MUHREC Approval CF16/763 - 2016000370 - Eliminate Dengue - Community based field releases of Wolbachia infected mosquitoes in Townsville, Queensland.

Surveys were undertaken under Monash ethics: MUHREC Approval CF13/2805 - 2013001515 - Eliminate Dengue - Community knowledge of dengue and Wolbachia based dengue control in Townsville, Queensland

Verbal and/or written consent from participants was obtained by phone, online or face-to-face to set BG traps, set MRCs (phase 1), or participate in Community Mosquito Releases.

Ethical approval was not required to access non-identifiable dengue case notification data collected as part of routine disease surveillance.

## Results and discussion

Prior deployments of *Wolbachia* in Australia by the World Mosquito Program utilised a traditional individual informed-consent approach to obtaining community authorisation for the releases ^7^. While this approach was adequate for small deployments, it was not considered scalable for an entire city. We therefore developed a Public Acceptance Model (PAM) that proved highly effective in ensuring community awareness and acceptance of the mosquito deployment program in Townsville. We believe this model will be suitable for other settings with appropriate local adaptation, and provides a framework for scaled deployment of this type of intervention globally.

Releases of mosquitoes in Townsville began in Oct 2014 with strong community support ( Table 2) and lasted for 28 months. The release program was divided into 4 sequential stages. The approach used in Townsville relied on the use of Mosquito Release Containers (MRC) as the preferred method of deployment ( Figure 2). In each suburb of the city MRCs were set at approximately 20% of residences and then refreshed with new food, water and eggs every 2–3 weeks. MRC release cycles continued until 2 consecutive samples of adult mosquitoes taken from the suburb showed a *Wolbachia* frequency above 50%; *Wolbachia* frequency in these areas was then monitored without additional releases. While the city occupies a municipal area of 190km ^2^, releases were undertaken over a reduced area of ∼66km ^2^ as not all areas of the greater municipal area were inhabited or provided suitable *Ae. aegypti* habitat ( Figure 1). The targeted release areas covered all of the suburbs where local dengue transmission had occurred during the prior 10 years and known high-risk suburbs for dengue transmission were targeted in stage 1. *Wolbachia* monitoring was conducted and infection frequency reported aggregate to suburb boundaries, encompassing an area greater than the 66km ^2^ of actual release areas. The total area considered ‘covered’ by *Wolbachia* in Townsville is 128km ^2^, with a residential population in 2016 of 140,000.

*Wolbachia* establishment across the different suburbs of Townsville for the four stages is shown in Figure 3. In general, establishment of *Wolbachia* occurred reliably after releases stopped once the 50% threshold was met. In some suburbs, *Wolbachia* frequencies fluctuated for a number of months before eventually rising to above 80%. In five suburbs, a small number of supplementary releases were undertaken to ensure establishment. In all suburbs, the infection frequency has remained stable without any signs of *Wolbachia* being lost from the mosquito population ( Figure 3).

Laboratory experiments have suggested that maternal transmission of *w*Mel can become unstable in *Ae. aegypti* at high temperatures and plausibly might limit the field usefulness of the *w*Mel strain ^17^. The temperatures used in these incubator experiments were meant to mimic larval rearing temperatures in north Queensland. However, our field data shows long-term stability of *w*Mel, presumably because temperatures used in this study were not truly representative of those experienced by mosquitoes in the field. We assume that mosquitoes predictably seek out non-stressful microhabitat when it exists ^18^ and larval rearing temperatures do not mirror measured ambient temperatures. Empirical data from this study and other sites ^9^ suggests that *w*Mel is much more robust to deployment than predicted by 17.

A key feature of using MRCs for mosquito releases is the possibility of mobilising the community to undertake the deployment instead of employed program staff. In stage 1 of the release program staff undertook the deployment by setting and maintaining MRC buckets themselves. In stages 2–4 we used a blended approach of community members setting their own MRCs and then program staff members supplementing these deployments by distributing additional MRCs to meet the target of 20% of residences, to ensure adequate coverage without major spatial gaps. Community-based releases were undertaken in three ways; school programs where students were given MRC kits to take home, direct community releases where MRC kits were given to householders who had signed up to participate through community engagement activities, and finally by having large employers within the city distribute MRCs to staff who were willing to participate. Of the three methods, providing MRCs directly to the community was the most cost effective. It also allowed for more targeted deployment and better coordination with field staff, ensuring adequate coverage across a suburb. This blended approach of community-based deployment supplemented with programmatic targeted deployment is considered the most appropriate for future large-scale operations. The schools program – while being less efficient and costlier – proved to be an excellent community engagement vehicle, with the release outcome of secondary importance. Its success was highly dependent on working with an actively engaged teacher who could serve as a champion for the program.

**Figure 4.**
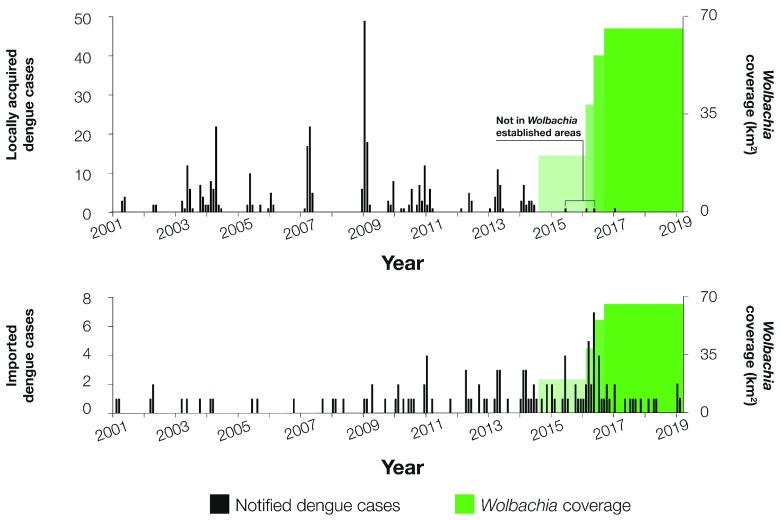
Dengue case notifications. Dengue case notifications per month in Townsville, Australia, January 2001 – March 2019, before and after *Wolbachia* mosquito deployments. Notifications include laboratory-confirmed and probable dengue cases, classified as locally-acquired ( **A**) or imported ( **B**) based on a history of overseas travel to a dengue-affected country during the period 3 – 12 days prior to illness onset. Data was extracted from the Queensland Health Notifiable Conditions System (NOCS) on 11 April 2019. Green shading shows the four stages of *Wolbachia* deployment conducted in Townsville since October 2014.

Episodic outbreaks of locally transmitted dengue have occurred annually in Townsville since 2001. Outbreaks occur against a background of regular importations of dengue into Townsville by international travellers ( Figure 4). In the period since *Wolbachia* deployments began in Townsville in 2014, dengue case importations have continued to occur, with 54 imported cases in the 53 months from November 2014 – March 2019 compared to 41 in the preceding 53-month period. Notably, only four locally-acquired dengue cases have been identified in the post-release period, compared to 94 in the equivalent preceding period and a median of 131 (IQR 101-143) in all 53-month moving windows since 2001. In none of the previous 53-month moving windows since 2001 were there fewer than 69 locally-acquired cases notified. Importantly, only one of the four local cases since November 2014 was resident in an area where *Wolbachia* had been established. However, public health investigation found that this case was highly mobile and therefore the likely place of acquisition was uncertain. The model-based estimate of intervention effect from the interrupted time series analysis suggests a 95% reduction in dengue incidence in *Wolbachia* treated populations (95% confidence interval: 84–98%), adjusted for season, imported cases, and allowing for temporal autocorrelation of cases ( Table 5). These findings, coupled with continuous validation of the impaired vector competence of *w*Mel-infected *Ae. aegypti* in release areas ^19^, represent empirical epidemiological evidence consistent with modelling projections of *w*Mel-mediated elimination of dengue transmission in most settings ^6^.

The cost of undertaking the program per person, and per km ^2^, varied between stages, and when time to complete each stage was also considered stage 2 was most efficient ( Table 3). Considering the low population density of this city we expect the cost per person, for the same deployment methodology, would be dramatically reduced in many tropical cities with much higher population densities. Furthermore, the costs for the deployment in Townsville were inflated as the work was undertaken as a research activity, with much more monitoring than would be expected in an operational public health intervention. The breakdown of costs by major activity are shown in Table 4. Community engagement activities accounted for a significant part of the cost of deployment, which shows the prioritization of and importance given to these activities by the World Mosquito Program. This, together with the cost of deployment (staff, vehicles etc.), accounted for more than half the cost of the implementation, and represents the areas where significant cost reductions might occur in future operational deployments. Given the costs for this study, and considering that future deployments should utilize less monitoring and occur in settings of higher population density, we estimate that deployment cost should be able to be reduced to less than US$1 per person. Additionally, in contrast to most other interventions, this cost should not be ongoing since once *Wolbachia* is introduced it is expected to maintain itself in populations. This suggests that the use of *Wolbachia* for arbovirus control as described in this study has the potential to be an extremely cost effective intervention compared with existing methods and many other proposed interventions that feature the release of modified mosquitoes ^10^.

**Table 3.** Cost per person and cost per km ^2^ for each of the four release stages in Townsville.

Stage	Release area km ^2^	Months required to deploy	Average FTE ^1^	Cost per person AUD$	Cost per km ^2^ AUD$
Stage 1	20.3	14	10	29	69,762
Stage 2	18.2	6	12	16	37,268
Stage 3	17.6	4	11	19	23,231
Stage 4	9.7	5	8	13	37,313

^1^Average number of full-time equivalent (FTE) staff used to undertake deployment. It excludes staff required to produce mosquitoes for release or undertake diagnostics.

**Table 4.** Costs by major activity class for entire deployment.

Expense category	% of total costs	Major cost components
Community Engagement	23	Staff, surveys, advertising & media, events, catering, overheads
Field Deployment	41	Staff, transport, equipment, MRCs, overheads
Monitoring	24	Staff, transport, BGS traps, GIS, supplies, overheads
Diagnostics	9	Staff, reagents
Production	2	Staff, consumables

MRC - mosquito release containers, BGS- Biogents Sentinel, GIS- Geographic Information Systems

**Table 5.** Model estimates from negative binomial regression of monthly locally-acquired dengue case counts in *Wolbachia*-treated vs untreated populations, adjusted for temporal autocorrelation within each transmission season.

Variable	IRR	95% CI	Robust SE	p value
*Wolbachia* intervention (treated vs untreated)	0.05	0.02 – 0.16	0.03	<0.001
Season (dry vs wet)	0.27	0.10 – 0.50	0.09	<0.001
Monthly imported dengue cases (≥1 vs 0)	2.21	1.08 – 4.54	0.81	0.031

IRR: incidence rate ratio; CI: confidence interval; SE: standard error.

 This study demonstrates that: the *w*Mel strain of *Wolbachia* can be deployed effectively across large geographic areas at low cost; that once the intervention is deployed it is stable and self-sustaining; and that communities are accepting of the release of mosquitoes and are willing to participate in deployments when effectively engaged. From this study, we were able to identify a number of key learnings to take into future studies. These include: the understanding that community engagement approaches can be successfully scaled without compromising their quality, that shipping eggs from a remote production facility is possible but that care is needed with the shipping method to avoid excessive mortality, that managing egg strips for quality and to estimate quantity was laborious and a key step to improve in future scale-up. Finally, a time series analysis of notified dengue cases within the city over a 18-year period is consistent with modelling predictions of a large impact on dengue transmission ^6^ – and indeed in this city the observational data is consistent with elimination of local transmission.

## Data availability

The data underlying Figure 3 is available from Figshare.

O'Neill, Scott (2019): Graph Data Version 3. figshare. Dataset.

https://doi.org/10.6084/m9.figshare.8282306.v1 ^20^.

This dataset is available under a CCO license.

Human dengue case notification data was provided to us by Queensland Health. The conditions of release of the raw dengue case notifications data to us by the Communicable Disease Branch of Queensland Health do not permit further sharing to a third party. This data (local and acquired dengue case notifications from Townsville local government area, Jan 2001 - June 2018) can be acquired by application to Queensland Health:

https://www.health.qld.gov.au/clinical-practice/guidelines-procedures/diseases-infection/surveillance/reports/notifiable/data-request
