# Supplementary material for: Scaled deployment of Wolbachia to protect the community from dengue and other  Aedes transmitted arboviruses
Source: Gates Open Res. 2019 Aug 13;2:36. Originally published 2018 Aug 1. [Version 3] doi: 10.12688/gatesopenres.12844.3 (PMC6305154; doi:10.12688/gatesopenres.12844.3)
Supplement: Peer review history

# Peer review: Reviewer response for version 2

**DOI:** 10.21956/gatesopenres.13975.r26733

**Published:** 2018-11-02

**Authors:** Horacio M. Frydman, Heverton Leandro Carneiro Dutra

**Affiliations:** 1 Department of Biology, National Emerging Infectious Diseases Laboratories (NEIDL), Boston University, Boston, MA, USA; 2 Department of Biology, Boston University, Boston, MA, USA

We consider that the authors have addressed our questions.

We confirm that we have read this submission and believe that we have an appropriate level of expertise to confirm that it is of an acceptable scientific standard.

# Peer review: Reviewer response for version 1

**DOI:** 10.21956/gatesopenres.13925.r26600

**Published:** 2018-08-28

**Authors:** Horacio M. Frydman, Heverton Leandro Carneiro Dutra

**Affiliations:** 1 Department of Biology, National Emerging Infectious Diseases Laboratories (NEIDL), Boston University, Boston, MA, USA; 2 Department of Biology, Boston University, Boston, MA, USA

**Overall comments:**

 The manuscript “Scaled deployment of *Wolbachia *to protect the community from *Aedes *transmitted arboviruses” reports a successful strategy for the long-term establishment of *Wolbachia *on a citywide scale. Previous pioneer work from this group have shown that *Aedes* *w*Mel *Wolbachia *strain can establish itself in the field after surpassing a certain unstable equilibrium threshold (Hoffmann et al., Nature 2011). However, in the current study, the establishment of *Wolbachia* is done at a larger scale, across 66 km ^2 ^of Townsville, a city in northern Australia with a population of approximately 187,000 residents. Interestingly enough, the authors also report details on a community engagement approach used to achieve *Wolbachia *establishment in a cost-effective manner. 

 Remarkably, the results presented here provide the first field-based evidence that the self-sustainable *Wolbachia* deployment strategy works against dengue. That being said, this study was not designed as a clinical trial experiment, as pointed out by the authors. Although extremely promising and exciting, the results presented here should not be taken, at least at this point, as a definitive evidence that *Wolbachia *will block dengue transmission in endemic areas. Townsville, has a relatively low number of dengue cases a year, compared to a great number of cities in tropical and sub-tropical areas of the globe. On that regard, this non-profitable consortium has ongoing field-trials in places like South America and Asia, serious contenders to the *Wolbachia*-based strategy.

**Major comments: **

1. The reader is left wondering if non- *Wolbachia *factors in Townsville (e.g. climate) are contributing to the remarkable decrease of reported locally acquired dengue cases after *Wolbachia *release (2016-2018). This work would greatly benefit from providing additional epidemiological data. Showing the dengue cases for other cities in Northern Australia with no *Wolbachia *release over the same period as shown in Figure 4 (2002-2018). This data could be incorporated in Figure 4 as an additional histogram. Ideally this histogram should show an average of locally acquired dengue case notifications from 2002 to 2018 from several cities with epidemiology similar to Townsville from 2002 to 20014 (prior to *Wolbachia *release). Although not a rigorous control, this data would at least give some indication if non- *Wolbachia *factors in that region (may be climate?) could be playing a role for the dengue decrease. 
  (note added in proof: the other reviewer also had a somewhat similar comment). 
2. A small discussion of the challenges faced by their approach as well as lessons learned from Townsville that should be considered in areas were *Wolbachia *deployment is imminent would be valuable to the field. 

 

**Minor comments and suggestions by page**

**Main Text: **Original text in *italics*

**P.1**: Title: *Scaled deployment of Wolbachia to protect the community from Aedes transmitted arboviruses*

 Although there are several studies demonstrating efficient pathogen blocking effect of *Wolbachia *against several arboviruses transmitted by *Aedes aegypti*, including many from this research group, this specific manuscript only shows epidemiological data on dengue. Therefore, the title would be more precise if changed to reflect the specific arbovirus evaluated. 

**P.4**: Rearing (2 ^nd^paragraph): “ *The wildtype colony was backcrossed for three generations*…”

 Although there is data pointing towards the inexistence of *kdr *mutations in the local *Ae. aegypti *population present in Queensland, indicating susceptibility to pyrethroid insecticides, there are several recent reports indicating the sporadic detection of non-native *Ae. aegypti *mosquitoes carrying insecticide resistance alleles not found in Australia. Areas with intense international flux like seaports and airports are a point-of-entry for these alleles into the local population (Endersby-Harshman *et al*., 2017 ^1^). How is the WMP taking the potential risk of insecticide resistance into consideration when rearing their mosquitoes for field releases? Few sentences regarding this aspect would be helpful, given that insecticide resistance alleles could highjacks *Wolbachia* establishment in areas heavily treated with insecticides by indoor residual spraying (IRS). Are the mosquitoes selected for backcrossing checked for chemical compounds resistance? What is the level of synchrony between the WMP approach and the guidelines established by the Queensland dengue management plan 2015-2020 which indicates the use of IRS as an approach against disease vectors?

**P.4**: Rearing (2 ^nd^paragraph): “ *In order to prevent inbreeding, 10% wildtype males were added to each generation of the ‘broodstock’*.”

 To avoid inbreeding is of great relevance. That being said, is this value of 10% resulted from a pool of males collected across all the same 49 sites in Townsville, the same used to establish the original wildtype colony? Additionally, it is to be expected that at a certain point, given the establishment of *Wolbachia* in the field, that the males collected would harbor the bacterium. Did the authors screened a portion of the males added to the cages after the copulation period?

**P.5**: Rearing (7 ^th^paragraph): “ *Wolbachia* *infection was also tested each week of production, 80 females and 80 males were screened from each broodstock cohort*…” 

1. These numbers of female and males tested represents what percentage of the total population? 
2. Given the potential for maternal transmission leakage of *Wolbachia *when considering the transmission dynamics of this bacterium, something speculated to be one of the factors contributing to the difficult establishment of *Wolbachia *in Cairns, another area where releases by the WMP took place (Schmidt *et al*., 2018 ^2^), it is somewhat unexpected that the field colony was not screened prior to release, only the broodstock. What would be the reason for that? May be the consistency by which the mosquitoes were reared under laboratory conditions?

 
**P.5**: Rearing (8 ^th^paragraph): “ *The colony was therefore refreshed with 2500 males and 2500 females each week*.”

 The same question previously asked ( **P.4**: Rearing (2 ^nd^paragraph). Are these mosquitoes obtained from a pool across all collection sites or derived from a single site? 

**P.5**: Rearing (8 ^th^paragraph): “ *This was achieved by placing suspected male pupae based on size into cups of 10;*…”

 Given the difficulty of visually sexing each pupae cup without the aid of software and hardware-based engineering, what was the overall confidence level in this sex by size strategy? Although there is no risk for the community, as the CI and female-based deployment deals with the issues associated with accidental female release in this case, it would be interesting to address this aspect of the method used by the research team. 

**P.7**: Mosquito releases (3 ^rd^paragraph): “ *For stage 1, it required between 7 and 19 weeks of releases for each suburb to reach that target*.”    

 It is known that the rate of dispersion of *Ae. aegypti *correlates with the human density in a given area. As such, how long did the authors wait to start screening field collected mosquitoes, as a way to avoid screening the same mosquitoes that were released in a particular area? It is not clear how far the BG traps were set apart from the MRC’s. 

**P.7**: Mosquito releases (2 ^nd^and 4 ^th^paragraphs): In summary, MRC’s had 100 eggs / 1L of water and 5 (summer) / 6 (winter) wafers of Aqua One vege wafer fish food, while Mozzie boxes had 100 eggs/ 400mL of water and 4 (summer) / 5 (winter) wafers of Aqua One vege wafer fish food. In terms of quality assessment of the fitness of the mosquitoes being released, how was the comparison between the MRC’s and the Mozzie boxes?

**P.7**: Mosquito releases (5 ^th^paragraph): “ *Finally, in the last two suburbs of stage 3 (Kelso & Rasmussen) and across stage 4, releases of adult mosquitoes were used to fill in gaps in MRC coverage*”.

 Here two distinct deployment strategies were combined. As such, the release procedures used were the same as established in Hoffmann *et al*., 2011 (citation #7), in terms of number of females and release period (in weeks)? How this combined approach compares to the deployment of *Wolbachia *through a single release method in terms of establishment efficiency? 

**P.8**: School releases (2 ^nd^paragraph): “ *…but made of clear plastic to encourage student observation (Figure 2B)*”.

 No suggestion here. Just a praise to the attention to the details contributing to the community engagement. WMP is not only deploying their method, but also educating the community. 

**P.8**: School releases (3 ^rd^paragraph): “ *…who gave presentations encouraging participation prior to each of the three mosquito release cycles*.”

 How was the level of engagement along the three cycles? Given the author’s interesting approach, this information could be helpful as a proxy for the predicted efficacy of this strategy in release areas worldwide.

**P.12**: Results and discussion (3 ^rd^paragraph): “ *In general, establishment of Wolbachia  occurred reliably after releases stopped once the 50% threshold was met.*”

 To date, does the WMP continues to screen these areas where *Wolbachia* was released roughly 2-3 years ago? If the answer is yes, does the infection frequency still high in these areas? I am particularly interested in the Condo area, where the last data point shows an infection frequency of 51.79%.

**Final thoughts and suggestions:**

 The following questions are not within the scope of what is proposed for this manuscript, just a couple suggestions.  

1. Have the authors considered screening areas where *Wolbachia *deployment did not occur but were adjacent to release zones? For instance, given the low human density in Townsville, a key factor associated to mosquito dispersal, as previously stated, and recent data showing challenges in *Wolbachia *establishment in Cairns, having long-range dispersal as one potential reason, would be interesting to see how contained *Wolbachia* deployment is, in my opinion this information would be particularly relevant in areas where *Wolbachia *is soon to be deployed, as it can avoid legal conflicts within the context of community acceptance in neighborhoods where the use of the bacterium has not been yet approved or assessed. 
2. Would be interesting to see data based on mitochondrial DNA screening in *Wolbachia*-harboring mosquitoes. The fine-tune of such approach using Townsville as a model would benefit the program as a whole, providing information related to mosquito immigration events and potential imperfect transmission of the bacterium. 
3. A future genomic analysis of the mosquito population pre- and post- release of *Wolbachia *would provide a unique opportunity to evaluate the intrinsic impact of the bacterium on the mosquito population structure as a whole.

 

 Finally, I want to take this opportunity to congratulate the authors and the team personnel for the massive work necessary to obtain the results described here.

We confirm that we have read this submission and believe that we have an appropriate level of expertise to confirm that it is of an acceptable scientific standard, however we have significant reservations, as outlined above.

## References

[1] : Pyrethroid Susceptibility Has Been Maintained in the Dengue Vector, Aedes aegypti (Diptera: Culicidae), in Queensland, Australia. *J Med Entomol*.2017;54(6) : 10.1093/jme/tjx145 1649-1658 10.1093/jme/tjx145 28981684

[2] : Fine-scale landscape genomics helps explain the slow spatial spread of Wolbachia through the Aedes aegypti population in Cairns, Australia. *Heredity (Edinb)*.2018;120(5) : 10.1038/s41437-017-0039-9 386-395 10.1038/s41437-017-0039-9 29358725PMC5889405

# Peer review: Reviewer response for version 1

**DOI:** 10.21956/gatesopenres.13925.r26599

**Published:** 2018-08-10

**Authors:** Gregor J. Devine

**Affiliations:** 1 Mosquito Control Laboratory (MCL), QIMR Berghofer Medical Research Institute, Herston, Qld, Australia

**Summary:**

 Wolbachia-mediated control is one of the most exciting recent developments in the struggle against dengue and other mosquito borne diseases. We already know from this group’s previous work in Cairns that the replacement of wild-type *Aedes aegypti* populations with Wolbachia-infected forms is both feasible and sustainable in towns of around 150,000 people.

 Epidemiologically, it would have been more interesting to see a summary of impacts on dengue transmission in Cairns as, historically, that town experiences far more dengue transmission and more dengue imports than Townsville.

 The novelty in the current report is that this is the first “citywide scaled deployment”. This scaling refers to the direct involvement of the community and local school children in executing the releases.

**Comments:**

 I would have liked a little more background and explanation on the following: 

1. What is the rationale for backcrossing the wMel strain with a local wildtype for three generations prior to release? Is there any underlying empirical basis for this in terms of fitness and/or genetic homogeneity?
2. It appears from Fig 3 that, by the end of the monitoring period (mid 2016), very few mosquitoes were being captured and that almost all were Wolbachia-infected. Was that very low density a result of a hostile climate? Is it possible that mosquito suppression as well as replacement is having an impact on transmission here? Is declining mosquito density a feature of wMel establishment? Were any Aedes endemic, non-release areas monitored for comparison? 
3. The authors state that the outcomes of releases by schools-based programs were of secondary importance to their value as instruments of engagement. In this paper, it is flagged as a major component of “scaled deployment” so it would have been interesting to report on the operational success of the schools-based program. Was there any evaluation of compliance (observation of the releases made by children) or of the rates of Wolbachia replacement in areas specifically targeted by schools?
4. It seems likely that Wolbachia is reducing transmission risks in Townsville, but other contributing factors may be being ignored. My understanding is that releases of Wolbachia in Townsville have coincided with some of the hottest and driest years on record. Climate has a direct impact on mosquito survival and is strongly correlated with transmission. 
5. There’s no real discussion of limitations or challenges here. The authors state that economies of scale, in regions of high population density, will result in successful deployments costing less than $1 per capita. The assumption is that population replacement and dengue-blocking across hyperendemic urban sprawls will be cheap and simple.   
  Townsville has very limited dengue transmission and adult indices appear lower than for many tropical cities. Does the WMP not see some issues with extrapolating successes in Townsville to the rest of the world?
6. The authors dismiss work on Wolbachia loss and heat stress (Ulrich et al PLoS NTDs, Ross et al PLoS Pathogens) but Aedes do demonstrate fast and successful development in the field at water temperatures ≥ 30 ^o^C and the truth is that we don’t know much about the operational impacts of those conditions on Wolbachia stability. 
  This paper certainly suggests that, in Townsville, Wolbachia-infection is stable. But in Townsville monthly average high temperatures range between 31.5 and 25.1 ^o^C. In Bangkok, they range from 35.4 to 31.7 ^o^C. A global Wolbachia operation will at least need to consider the potential impacts of high water temperatures in aquatic habitats.
7. What are the factors that result in a minority of sites hovering around 70% Wolbachia coverage? Is it immigration, or the presence of some protected, wild-type reservoir. Or is it related to mosquito density and a relatively small number of dispersal events? 

Overall, this paper represents a keenly awaited progress report on the stability and feasibility of Wolbachia releases in Australia and the ways in which those releases might be made more cost effective.

I confirm that I have read this submission and believe that I have an appropriate level of expertise to confirm that it is of an acceptable scientific standard.

# Response

**Published:** 2018-08-16

**Authors:** Scott O'Neill

**Affiliations:** Monash University, Australia

**Response to reviewer 1**

 The reviewer’s statements around novelty are incorrect in stating that we already know that we can deploy in cities of 150,000 by referring to deployments around Cairns, Australia. The only studies published from Cairns relate to small pilot deployments. Many small pilots have been undertaken in Cairns and surrounding areas since 2011 that have focused on obtaining data on the best ways to deploy mosquitoes. In the last 2 years, we have “filled in” around these pilot areas to provide broad coverage to all of North Queesnland’s major dengue risk areas. This study is actually the first published study of a citywide deployment being undertaken as a single project which shows that a “greenfield” city can be engaged and the intervention deployed at scale, cost effectively and quickly. The use of the community to deploy is an additional component of the paper but not the primary aim.

 We have a companion paper in preparation that will report the results from deployments in the city of Cairns that will reinforce the findings of this study.

**Specific Comments**

 1. We undertook three generations of backcrossing to make sure our release strain closely approximated the genetic background of the Townsville target population. This was done as a precautionary measure and was not based on specific empirical data that aimed to characterise any differences between the Cairns and Townsville genetic backgrounds of the resident mosquitoes.

 2. Figure 3 shows total counts of mosquitoes that were run through diagnostics in a given sampling period. This is presented to help provide an estimate of sample size that underpins a given frequency estimate. There is a correlation between this number and actual mosquitoes caught but it cannot be used to estimate population size because the number of traps contributing the sample was variable. We reduced BG traps in a given area after Wolbachia was considered established (often by more than 60%) and these traps were moved to areas where active releases were being undertaken. As a result, the impression that mosquito populations declined after release is not accurate. Modelling predicts that mosquito populations should reduce slightly after the introduction of Wolbachia but we did not attempt to measure that in this study

 3. Because the schools program used volunteer students to undertake the releases and these children were scattered geographically they were supplemented with programmatic releases in adjoining areas which made it impossible to compare their effectiveness from an entomological perspective with purely programmatic releases. We did undertake QA procedures on a sample of student release containers to evaluate the program. The major consideration for us was that this form of release was quite expensive compared to other forms so from a purely economic perspective it was inefficient. However, its value to us was more from a community engagement perspective as Schools are fundamentally trusted in the community and it was an indirect method to engage parents of school children through undertaking the releases and increase community awareness of the program.

 4. Temperatures between 2015-18 in Townsville have been above average and rainfall below average but certainly not the hottest or driest years on record. Indeed, if you examine the meteorological data for the period in which we analysed dengue cases you can see that similar climatic conditions were experienced in Townsville between 2001 -2006 and during these years significant local dengue transmission events occurred, indicating no obvious correlation between temperature/rainfall variability in Townsville and dengue transmission. See figure below for data

**Yearly rainfall (A), maximum temperature (B) and minimum temperature (C) anomalies, and locally acquired dengue cases (D) for Townsville from 2001-2018.** Yearly rainfall anomaly values were calculated by comparing yearly, northern wet season rainfall totals (cumulative rainfall totals from October to April each year) to the long-term, 30-year wet season yearly average (October 1961 to April 1991; Standard Reference Period as defined by World Meteorological Organisation) (Bureau of Meteorology, Retrieved  15 August 2018 from http://www.bom.gov.au/climate/how/newproducts/map-periods.shtml); yearly maximum temperature anomaly values were calculated by comparing yearly, northern wet season maximum temperature mean values (daily maximum temperatures from October to April) to the long-term, 30-year wet season average (October 1961 to April 1991); yearly minimum temperature anomaly values were calculated by comparing yearly, northern wet season minimum temperature mean values (daily minimum temperatures from October to April) to the long-term, 30-year wet season average (October 1961 to April 1991); yearly cumulative dengue cases from October in the previous year through to September in the current year (Note: 2001 cumulative cases from January to September 2001; 2018 cumulative cases from October 2017 to June 2018). Weather/climate data obtained from Bureau of Meteorology (Station: TOWNSVILLE AERO, 032040). Green shading represents period of *Wolbachia* mosquito release and establishment (commenced October 2014).

 5. The main assumption underlying reduction in costing in based on the fact that major urban tropical cities have much higher population densities than Australian cities but many of the costs in deployment relate to the area of deployment. This means that costs will automatically decline in areas of higher population density and that is what we have seen in other work of the WMP where deployments have already been undertaken successfully as part of clinical trials that are underway in other countries.

 6. Our criticism is that the heat stress experiments have been undertaken either in incubators or in semi-field settings and then the results extrapolated to infer a potential problem in stability of *w*Mel in the field. However, the field is much more complex than an incubator and many breeding sites are cryptic and difficult to assay, so the true water temperature experienced by a population of mosquitoes in the field cannot be accurately measured. What we do know from this and unpublished work in other cities is that *w*Mel appears unaffected in locations with ambient temperatures that might indicate problems for *Wolbachia* transmission based on these studies. We can only assume from this that the studies predicting breakdown of wMel are not actually predictive of field settings.

 7. Our current hypothesis is that *Aedes aegypti* can often have a relatively large number of eggs in a dried state in a given area and over time these eggs will hatch depending of factors such as rainfall events or human assisted wetting. This mimics the effect of *Wolbachia* uninfected individuals immigrating into an area. Over time this egg bank becomes depleted and frequencies then move to stable rates closer to 90% or above.

# Response

**Published:** 2018-08-17

**Authors:** Scott O'Neill

**Affiliations:** Monash University, Australia

For access to temp and rainfall data in Townsville see

https://s3-eu-west-1.amazonaws.com/gatesopenresearch/linked/180566.0%27NeillFigure.docx

# Response

**Published:** 2018-10-25

**Authors:** Scott O'Neill

**Affiliations:** Monash University, Australia

**Response to reviewer 2**

 Response to General comments

 1. Unfortunately similar work as reported here was being undertaken in other population centres in Australia at the same time and as a result there is no suitable site to use as an external control that is of equivalent size and not having Wolbachia mosquitoes released. We provided a detailed response to the other reviewer indicating that weather conditions across the monitoring period before and after releases did not correlate with the observed reduction in dengue cases.

 2. Discussion addressing this point has been added to the revision.

 Detailed points

 P1. Title changed to explicitly reference dengue

 P4. In releases in Australia our approach was just to backcross to local populations in order to attempt to match the local population in various traits including insecticide resistance. We did not do more than this in Australia since early pilot studies (eg Hoffmann et al 2011) showed that this strategy was adequate. As shown by the results in this setting this was a sufficient approach to obtain success. In other countries, we have had to develop different strategies to deal with high levels of insecticide resistance. These approaches and results will be presented in future papers describing results from those study sites. We feel that it would be better to address those topics in those papers rather than in this paper that did not examine insecticide resistance deeply.

 P4. Our collections of wild type material were made from pooling across many ovitraps placed in areas where Wolbachia had not been released as we did for the initial backcrossing. Our goal was to generate genetically diverse material so it was pooled. Given that we could store the eggs we were able to maintain material for outcrossing during the release period as described in methods. As a result, we did not monitor for Wolbachia in the males. In future studies, there is no reason to exclude already treated areas or for the included males not to be Wolbachia infected. We have added text to the revised paper to improve clarity.

 P5. Wolbachia infection testing – 1. We tested 160 individuals from each cohort. A cohort was on average around 7500 individuals so around 2% of individuals. The sample size of 160 individuals was determined from a prior power analysis.  2. We have seen no evidence for reduced maternal transmission in our mass production.

 P5 – Yes these mosquitoes were derived from the same material used to outcross the colony in our Melbourne labs as well as backcross.

 P7 – Screening started prior to releases starting and was undertaken weekly. MRCs had variable placement due to the dependence f volunteers to host MRCs. It was not possible with that system to designate fixed differences between MRC’s and BGs. However, if you examine the shape of the establishment curves you can see that in most suburbs catching back of release material was not a big issue. If it was then you would expect to see Wolbachia frequencies climb quickly and then potentially decay after releases stopped. We only see those patterns in areas where adult releases took place.

 P7 – No fitness comparisons were made between the two but regular QA procedures indicated that emergence rates were similar between the two.

 P7 – The mixed approach used in the final experiments was undertaken due to operational issues with the completion of the projects and staff contracts completing. This was done to complete the study in time to coincide with staffing contracts finishing and was not set up as a comparison – it can’t really be analysed that way as a result. Comparisons between deployment methods are being undertaken in other sites in a more rigorous manner and these will be able to report on this comparison.

 P8 – Generally we found difficulty in sustaining interest in repeating the releases by the third round and our engagement officers needed to work harder to maintain interest levels in students for later round releases. As indicated in the paper we felt that school releases were better primarily as an engagement tool and direct public releases more effective from an establishment and cost perspective.

 P12 we have updated the figures with the latest monitoring data across the city to provide a more recent view of establishment success and dengue cases. Later monitoring has involved LAMP diagnostics and this protocol has also been included in the methods description. We have also updated the epidemiological figure with the latest data showing an extension of post release monitoring till Oct 2018 and continued impact on locally acquired dengue cases.

 Final thoughts

 1. Work on dispersal is underway in our Indonesian RCT site which will provide detailed information on Wolbachia dispersal between adjacent geographic areas. In generally natural dispersal rates are low as described already in Cairns.

 2. mtDNA as a marker for imperfect transmission or immigration cannot be done in Australia as the release strain carries the same mtDNA genotype as the wild population. It can be done however in other countries and is being done in different sites.

 3. Agreed and something for future work.
